# Supplementary material for: Yiai Fuzheng decoction inhibits triple-negative breast cancer by remodeling the immune microenvironment
Source: Front Immunol. 2025 Sep 30;16:1615631. doi: 10.3389/fimmu.2025.1615631 (PMC12518410; doi:10.3389/fimmu.2025.1615631)
Supplement: Supplementary file 2 [file DataSheet1.zip › Supplementary File 2/Treatment/C_vs_D/02_PCA/All_Groups/PCA_Score_2D_Label.pdf]

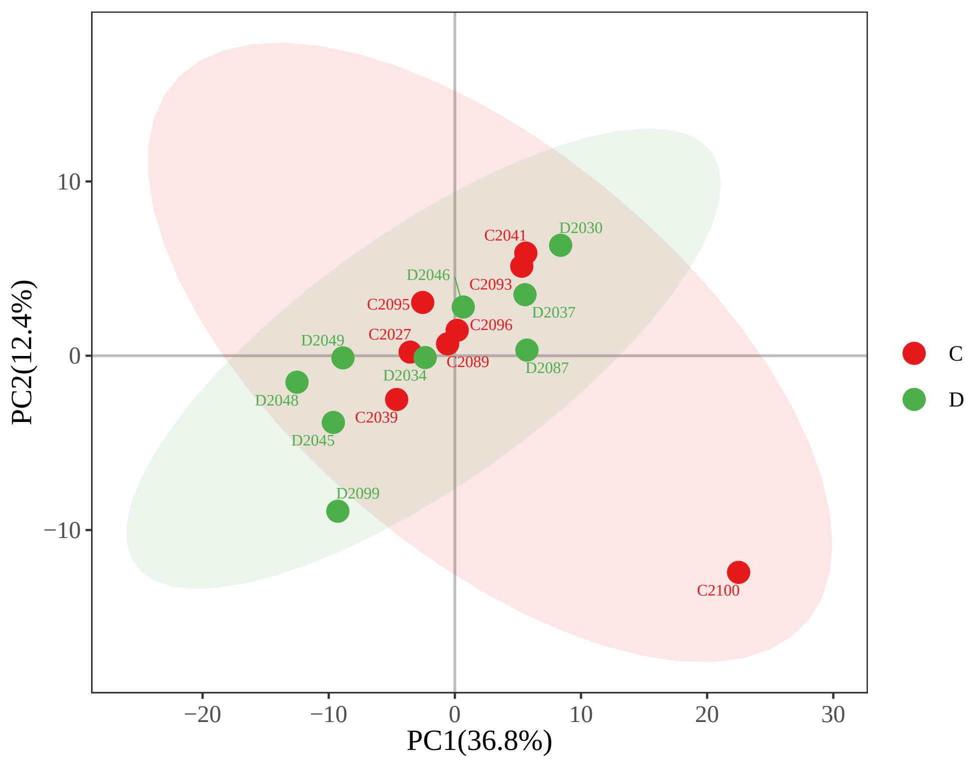

C2041
D2030
D2046
C2093
C2095
D2037
C2096
C2027
D2049
C2089
D2087
D2034
D2048
C2039
D2045
D2099
C2100
10
0
−10
−20
−10
0
10
20
30
PC1(36.8%)
PC2(12.4%)
C
D
